# The Role of Dual Mutations G347E and E349D of the Pigeon Paramyxovirus Type 1 Hemagglutinin–Neuraminidase Protein In Vitro and In Vivo

**DOI:** 10.3390/vetsci11120592

**Published:** 2024-11-25

**Authors:** Yu Chen, Junhao Gong, Tiansong Zhan, Mingzhan Wang, Shunlin Hu, Xiufan Liu

**Affiliations:** 1 Animal Infectious Disease Laboratory, College of Veterinary Medicine, Yangzhou University, Yangzhou 225012, China; 2 Jiangsu Co-Innovation Center for Prevention and Control of Important Animal Infectious Diseases and Zoonosis, Yangzhou University, Yangzhou 225012, China; 3 Jiangsu Key Laboratory of Zoonosis, Yangzhou University, Yangzhou 225012, China

**Keywords:** pigeon ND, PPMV-1, HN protein, pathogenicity, cross-species transmission, antigenic variation

## Abstract

**Simple Summary:**

Pigeon Newcastle disease is a prevalent viral infection in pigeons caused by pigeon paramyxovirus type 1 (PPMV-1), a variant of chicken-origin Newcastle disease virus (NDV). Key amino acid differences at positions 347 and 349 in the hemagglutinin–neuraminidase protein distinguish PPMV-1 from chicken-origin NDV. By introducing mutations at these positions, a recombinant virus, NT-10-G347E/E349D, was created in the present study. This mutant virus shows reduced replication and pathogenicity in pigeons but increased infectivity and transmission in chickens. The study highlights the role of these amino acids in the virus’s adaptation, offering insights into preventing and controlling PPMV-1 outbreaks.

**Abstract:**

Pigeon Newcastle disease (ND) is the most common viral infectious disease in the pigeon industry, caused by pigeon paramyxovirus type 1 (PPMV-1), a variant of chicken-origin Newcastle disease virus (NDV). Previous studies have identified significant amino acid differences between PPMV-1 and chicken-origin NDV at positions 347 and 349 in the hemagglutinin–neuraminidase (HN) protein, with PPMV-1 predominantly exhibiting glycine (G) at position 347 and glutamic acid (E) at position 349, while most chicken-origin NDVs show E at position 347 and aspartic acid (D) at position 349. However, the impact of these amino acid substitutions remains unclear. In this study, we generated a recombinant virus, NT-10-G347E/E349D, by introducing the G347E and E349D dual mutations into a PPMV-1 strain NT-10 using reverse genetics. The biological characteristics of NT-10 and NT-10-G347E/E349D were compared both in vitro and in vivo. In vitro, the G347E and E349D dual mutations reduce NT-10′s replication and neuraminidase activity in pigeon embryo fibroblast (PEF) cells while enhancing both in chicken embryo fibroblast (CEF) cells. Additionally, these mutations decrease NT-10′s binding affinity to the α-2,6 sialic acid receptor while significantly increasing its affinity for the α-2,3 receptor. In vivo, NT-10-G347E/E349D exhibited reduced pathogenicity in pigeons but increased pathogenicity in chickens compared to the parental NT-10 strain. The mutations also reduced the pigeon-to-pigeon transmission of NT-10 but enhanced its transmission from pigeons to chickens. Notably, significant antigenic differences were observed between NT-10 and NT-10-G347E/E349D, as an inactivated vaccine based on NT-10 provided full protection against NT-10 challenge in immunized pigeons but only 67% mortality protection against NT-10-G347E/E349D. Overall, these findings underscore the critical role of amino acids at positions 347 and 349 in PPMV-1 infection, pathogenicity, and transmission, providing a theoretical foundation for the scientific prevention and control of PPMV-1.

## 1. Introduction

Newcastle disease (ND) is a highly significant viral infection that impacts poultry globally, leading to substantial economic losses due to its high morbidity and mortality rates. Since the first documented outbreak in Indonesia in 1926, the Newcastle disease virus (NDV) has been responsible for at least four panzootics and remains widespread across the world today [1]. The causative agent, Newcastle disease virus (NDV), also known as avian paramyxovirus type 1 (APMV-1), belongs to the genus *Avulavirus* within the family *Paramyxoviridae*. Its genome is composed of a single-stranded, negative-sense RNA encoding six main structural proteins: nucleocapsid (NP), phosphoprotein (P), matrix (M), fusion (F), hemagglutinin–neuraminidase (HN), and large polymerase (L) [2]. Additionally, two nonstructural proteins, V and W, are produced through RNA editing during P gene transcription [2,3]. According to the updated unified phylogenetic classification system, NDV is classified into two classes (class I and class II), with class I consisting of only one genotype (genotype 1) and class II containing twenty genotypes (I–XXI, excluding XV) [4]. NDV strains differ in their virulence, ranging from highly virulent strains causing severe outbreaks to avirulent strains resulting in asymptomatic infections. While class I viruses are generally non-virulent, several class II genotypes (such as II, III, VI, VII, and IX) are virulent and pose a significant threat to the poultry industry [5].

Genotype VI NDV, commonly referred to as pigeon paramyxovirus type 1 (PPMV-1), is a major cause of ND in domestic pigeons and doves. According to previous classification methods, genotype VI included both pigeon-origin NDV (PPMV-1) and some chicken-origin NDV strains [6]. With the updated classification system, these chicken-origin NDV strains have been reclassified into genotype XX, leaving genotype VI to exclusively encompass PPMV-1. Despite this reclassification, PPMV-1 and genotype XX strains remain the most closely related in terms of genetic distance among all NDV genotypes. PPMV-1 is considered an antigenic variant of chicken-origin NDV, particularly genotype XX strains [7]. Although primarily adapted to pigeons, PPMV-1 is capable of infecting a wide range of avian species, including chickens and wild birds [8,9,10,11]. While most PPMV-1 strains are non-pathogenic to chickens, some can cause ND outbreaks in chicken populations, with their virulence potentially increasing after several passages through chickens [12,13].

PPMV-1 was first identified in the late 1970s in the Middle East and quickly spread to Europe, where it caused widespread outbreaks among pigeons, contributing to the third global ND panzootic in the 1980s [14,15]. Despite efforts to control the virus through vaccination, PPMV-1 has become endemic in several regions worldwide, including China, where it continues to circulate and cause substantial economic losses in the pigeon farming industry [16,17,18,19,20]. In China, specific vaccines against PPMV-1 are not yet available. Instead, pigeon flocks are typically immunized with inactivated or live vaccines derived from chicken-origin genotype II strains, such as LaSota or its variant Clone-30 [21]. However, antigenic differences between these chicken-origin vaccines and pigeon-derived genotype VI PPMV-1 strains have been noted, which may contribute to the ongoing prevalence of PPMV-1 in vaccinated pigeon populations [22,23].

The HN protein of NDV is a key surface glycoprotein and one of the primary antigenic components of the virus. It facilitates receptor recognition by binding to sialic acid residues on host cell glycoconjugates and exhibits neuraminidase activity, which cleaves sialic acid from newly formed virions to prevent self-aggregation and facilitate viral budding [24]. In addition, HN has been shown to promote fusion through its interaction with the F protein [25,26]. Moreover, as a major antigenic protein, HN is a primary target for the host’s immune response and is a crucial component in NDV infection and vaccine-induced protective humoral immunity [27,28]. To date, at least seven distinct antigenic sites on the HN protein have been identified through monoclonal antibody-mediated mapping. Among these, only the epitope formed by amino acids 345–353 is a linear epitope, while the other six antigenic sites are conformational [29,30]. In addition to these antigenic sites, five receptor-binding regions on the HN protein have also been reported, specifically at amino acids 193–201, 345–351, 494, 513–521, and 569 [31]. These findings suggest that amino acids in the 345–353 region may play a crucial role in both the receptor-binding function and the antigenicity of NDV.

Notably, previous studies have revealed significant differences in the amino acid composition of the HN protein between PPMV-1 and chicken-origin NDV strains [18,32,33,34,35]. Specifically, at positions 347 and 349 of the HN protein, PPMV-1 predominantly exhibits glycine (G) and glutamic acid (E), respectively, while most chicken-origin NDV strains display E at position 347 and aspartic acid (D) at position 349. However, the precise impact of these amino acid changes on PPMV-1′s biological properties remains unclear.

Given the potential significance of these amino acid differences, this study aims to elucidate the role of the G347E and E349D substitutions in the HN protein of PPMV-1. Using reverse genetics, we generated a recombinant virus based on the PPMV-1 NT-10 strain, introducing the G347E and E349D dual mutations into the HN protein. We then compared the biological characteristics of the wild-type NT-10 strain and the mutant NT-10-G347E/E349D strain both in vitro and in vivo to assess the effects of these mutations on viral replication, pathogenicity, transmission, and antigenicity. The results of this study provide valuable insights into the molecular mechanisms underlying PPMV-1 infection and cross-species transmission. Moreover, understanding the impact of specific amino acid substitutions in the HN protein on the virus’s biological properties may contribute to the development of more effective vaccines and control strategies against PPMV-1 and other NDV variants.

## 2. Materials and Methods

### 2.1. Ethics Statement

This study was conducted in strict adherence to the guidelines outlined in the Guide for the Care and Use of Laboratory Animals issued by the Ministry of Science and Technology of the People’s Republic of China. The animal experiment protocols were approved by the Jiangsu Administrative Committee for Laboratory Animals (approval number: SYXK-SU-2021-0027) and adhered to the welfare and ethical standards established by the committee. All experiments involving NDV were performed in an animal biosafety level 3 (ABSL-3) facility (CNAS registration No. CNAS BL0015) at Yangzhou University, in full compliance with the institutional biosafety manual and under the supervision of the Institutional Biosafety Committee of Yangzhou University.

### 2.2. Animals, Eggs, Virus Strains and Cells

Four-week-old specific pathogen-free (SPF) chickens were purchased from Zhejiang Lihua Agricultural Technology Co., Ltd. (Hangzhou, China). One-day-old chicks were hatched in our laboratory from SPF embryonated chicken eggs. One-month-old White King pigeons were obtained from a pigeon farm in Yangzhou, Jiangsu Province, China. All birds were confirmed to be free of NDV-specific serum antibodies by hemagglutination inhibition (HI) assay. Nine-day-old SPF chicken embryos were also purchased from Zhejiang Lihua Agricultural Technology Co., Ltd. (Hangzhou, China). The NT-10 strain, isolated from a diseased pigeon in Jiangsu Province by our laboratory, was propagated in 9-day-old SPF chicken embryos through allantoic cavity inoculation. Chicken embryo fibroblasts (CEFs) and pigeon embryo fibroblasts (PEFs) were prepared and cultured according to protocols described in our previous study [36].

### 2.3. Amino Acid Substitution Analysis

A total of 136 NDV HN gene sequences were obtained from GenBank (as of October 2024), including 127 PPMV-1 strains and 9 genotype XX strains. Gene sequences were aligned using MEGA 11 software (version 11.0.13) to ensure sequence consistency and accuracy. Conservation analysis of amino acid residues at positions 345 to 353 was performed using WebLogo 3 online software (https://weblogo.threeplusone.com/, accessed on 15 May 2023) to visualize residue conservation and substitution patterns across strains [37,38].

### 2.4. Generation of NT-10-G347E/E349D Recombinant Virus

The NT-10-G347E/E349D full-length cDNA plasmid was constructed by introducing G347E and E349D dual mutations into the HN gene of the NT-10 full-length cDNA plasmid, following the protocol provided by the Mut Express II Fast Mutagenesis Kit V2 (Vazyme, Nanjing, China). The virus rescue of NT-10 and NT-10-G347E/E349D was performed as described in our previous study [39]. In brief, BSR cells were first infected with fowlpox virus encoding T7 RNA polymerase for 1h, followed by co-transfection with the full-length cDNA plasmids of NT-10 or NT-10-G347E/E349D, along with helper plasmids encoding the NP, P, and L proteins. Three days post transfection, the culture supernatants were collected and used to produce viral stocks by inoculating into 9-day-old SPF chicken embryos. Total RNA was extracted from NDV-positive allantoic fluid using Trizol reagent (Invitrogen, Shanghai, China). Successful viral rescue was confirmed by PCR and C (Tsingke, Nanjing, China).

### 2.5. Detection of EID_50_, MDT, and ICPI

The 50% chicken embryo infectious dose (EID_50_) of the virus stocks was determined by infecting 9-day-old SPF chicken embryos with serial dilutions of the virus and calculated using the Reed and Muench method [40]. Mean death time (MDT) was assessed by inoculating 9-day-old SPF chicken embryos with the virus and recording the time to embryo death, following standard assay protocol [41]. The intracerebral pathogenicity index (ICPI) was evaluated by inoculating day-old SPF chicks intracerebrally with the virus and monitoring survival over 8 days, as described in standard assay method [41].

### 2.6. Growth Kinetics Assay

The growth kinetics of NT-10 and NT-10-G347E/E349D were measured to evaluate their replication efficiency in CEF and PEF cells. Virus was inoculated at a multiplicity of infection (MOI) of 0.01 in CEF or PEF cells and supernatant samples were collected at specified time points (12, 24, 36, 48, and 72 h post-infection, hpi) and replaced with an equal volume of fresh medium after each collection. Virus titers were determined by limiting dilution in CEF cells, following the Reed and Muench method, and expressed as the 50% tissue culture infectious dose (TCID_50_) [40].

### 2.7. Sialic Acid Receptor-Binding Assay

To determine the receptor-binding specificity of NT-10 and NT-10-G347E/E349D, a solid-phase direct-binding assay was performed as previously described [42]. In brief, two types of biotinylated, chemically synthesized trisaccharides, α-2,3 glycans (3′SLN) and α-2,6 glycans (6′SLN) (GlycoTech, Shanghai, China), were added in descending concentrations to 96-well streptavidin-coated microtiter plates (Thermo Fisher, Beijing, China). After removing the glycan solution, the plates were subjected to a blocking and washing cycle, followed by incubation with a solution containing 2^7^ hemagglutination (HA) units of each virus. Next, the plates were treated with antiserum specific to each virus, followed by incubation with horseradish peroxidase (HRP)-conjugated rabbit anti-chicken IgY antibody (1:2000, Thermo Fisher, Beijing, China). After adding TMB substrate, the reaction was stopped with 1 mol/L H_2_SO_4_, and absorbance was measured at 450 nm using a microplate reader.

### 2.8. Construction and Transfection of the HN Protein Expression Plasmids

The HN gene was amplified from the cDNA of NT-10 and NT-10-G347E/E349D using the specific primers: Forward (F) 5′-CAAGCTGGCTAGCGTTTAAACTTAATGGGCTCCAAACCTCACACC-3′ and Reverse (R) 5′-CGGGTTTAAACGGGCCCTCTAGTCATGTTCTTGTAGTGGCTC-3′. The amplified HN genes were cloned into the *EcoR*I and *Xba*I sites of the PCAGGS-Flag-N vector (HonorGene, Changsha, China) using the ClonExpress Ultra One Step Cloning Kit V2 (Vazyme, Nanjing, China). The recombinant plasmids were confirmed by Sanger sequencing and designated as pCA-HN and pCA-HN-G347E/E349D. For transfection, CEF or PEF cells were transfected at 40% confluency with the indicated plasmids by the EL Transfection Reagent (TransGen, Nanjing, China) following the manufacturer’s instructions.

### 2.9. Western Blot Assay

To detect exogenous HN protein expression, CEF or PEF cells were transfected with the indicated plasmids. At 72 h post-transfection (hpt), cells were lysed in RIPA buffer (Beyotime, Nanjing, China). Lysates were centrifuged at 13,000× *g* for 15 min at 4 °C, and supernatants were collected. Protein concentrations were measured using a BCA assay kit (Beyotime, Nanjing, China). Equal protein amounts were loaded onto 10% SDS-PAGE gels, separated, and transferred to PVDF membranes (Beyotime, Nanjing, China). Membranes were blocked with 5% skimmed milk for 1 h at room temperature, then incubated overnight with an anti-Flag (1:2000 dilution, TransGen, Beijing, China) or anti-GAPDH mouse monoclonal antibody (1:5000 dilution, TransGen, Beijing, China). After washing with TBST, membranes were incubated with goat anti-mouse IgG (H + L) HRP-conjugated secondary antibody (1:5000 dilution, TransGen, Beijing, China) for 1 h at room temperature. Protein bands were visualized with the BeyoECL Moon reagent (Beyotime, Nanjing, China).

### 2.10. Neuraminidase (NA) Activity Assay

NA activity was measured using an NA assay kit (Beyotime, Nanjing, China) according to the manufacturer’s protocol. Briefly, CEF or PEF cells were transfected with 1 µg of plasmids expressing either the wild-type or mutated HN protein. Then, the cells were harvested by centrifugation at 24 hpt. Harvested cells were resuspended in assay buffer and incubated with the NA fluorogenic substrate at 37 °C for 30 min. NA activity was measured using an ELISA reader with excitation at 322 nm and emission at 450 nm. NA index values were normalized to the wild-type HN protein transfection group, set as 100%.

### 2.11. Pathogenicity Assessment in Pigeons and SPF Chickens

To evaluate the impact of G347E and E349D dual mutations on viral pathogenicity, one-month-old White King pigeons (*n* = 9) and four-week-old SPF chickens (*n* = 9) were intranasally inoculated with 100 μL containing 10^6^ EID_50_ of either NT-10 or NT-10-G347E/E349D. Six pigeons in each group were monitored daily for survival over a 14-day observation period, with survival curves generated using GraphPad Prism 8 software (version 8.0.2). At 5 days post infection (dpi), three birds per group were euthanized, and tissue samples from the lung, trachea, duodenum, and spleen were collected for histopathological examination. These samples were fixed in neutral formalin, dehydrated, embedded in paraffin, sectioned, baked, dewaxed, and stained with hematoxylin and eosin. Prepared pathological sections were then examined and photographed under a light microscope to observe histopathological changes.

### 2.12. Transmission Experiment

To assess the transmission potential of NT-10 and NT-10-G347E/E349D, ten pigeons were inoculated with 10^3^ EID_50_ of either virus. At 24 h post inoculation, the infected pigeons were separated into two groups, each housed with five additional pigeons or SPF chickens as contact birds. Cloacal swabs were collected from contact birds at specified intervals to monitor viral shedding. Cloacal swabs were collected from contact birds at specified intervals to monitor viral shedding. Swab samples were processed by scrubbing and centrifugation, and the resulting supernatant was inoculated into 9-day-old SPF chicken embryos. After 96 h, the allantoic fluid was collected and subjected to an HA assay. An HA titer ≥ 4 log2 was considered positive for viral shedding.

### 2.13. Cross-HI Test

The antigenic relationships between NT-10 and NT-10-G347E/E349D were evaluated using a cross-HI test, as previously described [43]. Antigenic similarity was assessed using the antigenic similarity coefficient (R), calculated as follows: $R=\sqrt{r1\times r2}$, where r1 represents titer of strain A with antiserum B/titer of strain A with antiserum A, and r2 represents the titer of strain B with antiserum A/titer of strain B with antiserum B. Interpretation of R values is as follows: 0.67 ≤ R ≤ 1.5, indicates no significant antigenic difference between the two viruses; 0.5 ≤ R ≤ 0.67 indicates a minor difference between the two viruses; and R < 0.5 indicates a major difference between the two virus strains.

### 2.14. Statistical Analysis

All statistical analyses were conducted using GraphPad Prism 8 software (San Diego, CA, USA) using one-way or two-way ANOVA tests, with *p* < 0.05 considered statistically significant.

## 3. Results

### 3.1. Amino Acid Substitution Analysis at Positions 347 and 349 in the HN Protein of PPMV-1 and Genotype XX NDV

**Figure 1:**
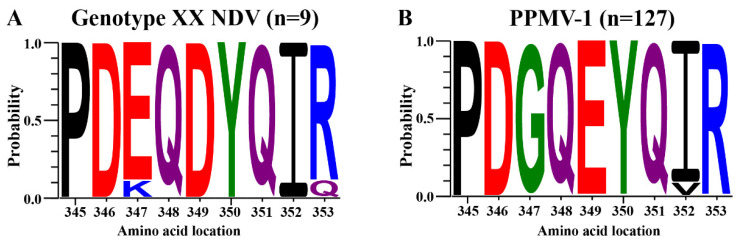
Conservation analysis of amino acid residues at positions 345 to 353 in the HN protein of PPMV-1 and genotype XX NDV. A total of 136 NDV HN gene sequences, including 127 PPMV-1 strains and 9 genotype XX NDV strains, were downloaded from the NCBI database. Conservation analysis of amino acid residues at positions 345 to 353 in the HN protein was conducted for genotype XX NDV (**A**) and PPMV-1 (**B**) using WebLogo 3 software.

Previous studies have indicated significant differences in the amino acid residues at positions 347 and 349 in the HN protein between PPMV-1 and genotype XX NDV [18,32,33,34,35]. To further validate these findings, we downloaded 136 NDV HN gene sequences from the NCBI database, covering the period from 2019 to 2024, comprising 127 PPMV-1 strains and 9 genotype XX NDV strains. A conservation analysis of amino acid residues from positions 345 to 353 was conducted using WebLogo 3 software (version 11.0.13) [37,38]. The analysis revealed that all PPMV-1 strains exhibited glycine (G) at position 347 (Figure 1B), whereas only one genotype XX strain had lysine (K) at this position, with the remaining genotype XX strains showing glutamic acid (E) (Figure 1A). Similarly, at position 349, all PPMV-1 strains consistently displayed E (Figure 1B), while all genotype XX strains exhibited aspartic acid (D) (Figure 1A). These results align with previous findings, confirming that specific substitutions—E347G and D349E—occur in the HN protein of PPMV-1 strains compared to genotype XX NDV strains.

### 3.2. Comparison of Pathogenicity and Transmission Abilities Between Three PPMV-1 Strains

To investigate the role of these specific substitutions, we first compared the infection and transmission abilities of three PPMV-1 strains isolated in our laboratory to select a parental strain with high replication and transmission efficiency. A total of twenty-four one-month-old White King pigeons, confirmed to be free of NDV HI antibodies, were randomly divided into four groups (*n* = 6). Each group was infected via intranasal routes with 100 μL containing 10^6^ EID_50_ of indicated virus or PBS as a control. At 12 hpi, four additional pigeons without NDV HI antibodies were added to each group as contact birds. Cloacal swabs were collected from inoculated and contact birds at designated days post infection to monitor virus shedding. As shown in Table 1, pigeons infected with the NT-10 strain exhibited higher mortality (5 deaths within 14 days) and a higher rate of virus shedding throughout the observation period compared to those infected with the JS/09/16/Pi and JS/07/04/Pi strains. Furthermore, at 11 dpi, all NT-10 contact birds (4/4) were shedding the virus, whereas only 50% of the contact birds in the JS/09/16/Pi and JS/07/04/Pi groups (2/4) showed virus shedding at 14 dpi. These findings suggest that NT-10 has stronger replication, pathogenicity, and transmission abilities in pigeons. Therefore, the NT-10 strain was selected as the parental strain for subsequent experiments.

### 3.3. Generation and Characterization of NT-10-G347E/E349D

The NT-10 strain was used as a backbone to generate a recombinant virus with G347E and E349D dual mutations using reverse genetics, named NT-10-G347E/E349D. The dual mutations and genetic stability across passages (P1–P5) were confirmed via PCR and Sanger sequencing We then compared the biological characteristics of NT-10 and NT-10-G347E/E349D, including EID_50_, MDT, and ICPI. As shown in Table 2, NT-10-G347E/E349D exhibited an EID_50_ of 10^7.83^/0.1 mL, compared to 10^7.0^/0.1 mL for NT-10, indicating that NT-10-G347E/E349D has a higher replication efficiency in chicken embryos. Additionally, NT-10-G347E/E349D displayed an MDT of 58 h and an ICPI of 1.51, consistent with a velogenic pathotype, while NT-10 had an MDT of 65 h and an ICPI of 1.34, classifying it as a mesogenic isolate. These results preliminarily indicate that the G347E and E349D dual mutations may enhance the replication ability of NT-10 in SPF chicken embryos and increase its pathogenicity in SPF chickens.

### 3.4. In Vitro Assessment of the Biological Characteristics of NT-10 and NT-10-G347E/E349D

**Figure 2:**
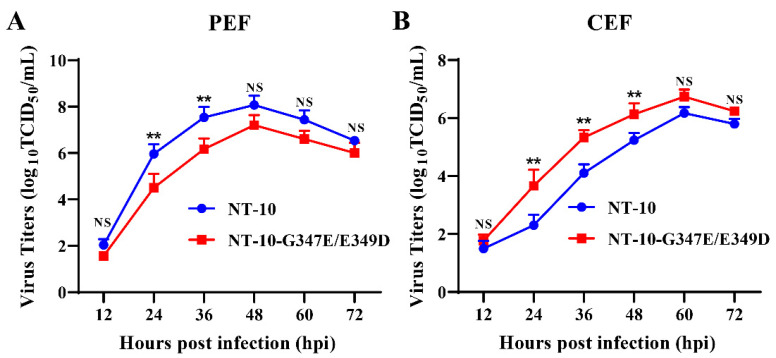
Growth kinetics of NT-10 and NT-10-G347E/E349D in PEF and CEF cells. PEF (**A**) or CEF (**B**) cells were seeded in 6-well plates at a density of 2 × 10⁵ cells per well and infected with the indicated virus at a multiplicity of infection (MOI) of 0.01. The infected cells were cultured in Dulbecco’s modified Eagle’s medium supplemented with 1% FBS at 37 °C under 5% CO_2_. Cellular supernatants were collected at indicated time points post-infection (12 h, 24 h, 36 h, 48 h, and 72 h), and viral titers were quantified as TCID_50_ using the Reed and Muench method. Error bars represent standard deviations (SDs) from triplicate analyses of three independent experiments. Statistical significance was assessed using a two-way ANOVA in GraphPad Prism 8 software. NS means no significant difference, ** *p* < 0.01.

To further examine the roles of amino acids at positions 347 and 349 in the HN protein, we compared the replication, receptor-binding and neuraminidase activities of NT-10 and NT-10-G347E/E349D in vitro. Viral replication activity was assessed through growth kinetics. As shown in Figure 2A, the virus titers of NT-10-G347E/E349D in PEF cells were consistently lower than those of NT-10 across all time points, with significant differences at 24 and 36 hpi. Conversely, in CEF cells, NT-10-G347E/E349D displayed higher titers than NT-10, with significant growth differences observed at 24, 36, and 48 hpi (Figure 2B).

**Figure 3:**
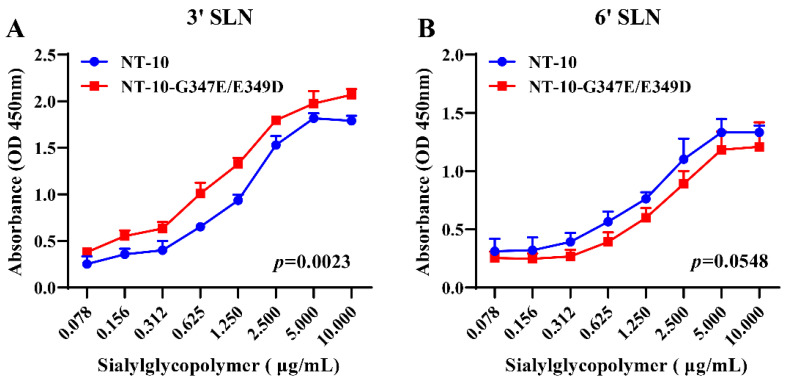
Sialic acid receptor-binding properties of NT-10 and NT-10-G347E/E349D. The direct binding of NT-10 and NT-10-G347E/E349D viruses to sialyl-glycopolymers containing varying concentrations of 3′SLN (α-2,3-linked sialic acids) (**A**) or 6′SLN (α-2,6-linked sialic acids) (**B**) was measured using a solid-phase receptor-binding assay. Viruses (2^7^ HA units) were incubated with 96-well streptavidin-coated plates coated with biotinylated glycans at descending concentrations for 2 h at 4 °C. Post-incubation, plates were treated with antiserum specific to each virus, followed by horseradish peroxidase (HRP)-conjugated rabbit anti-chicken IgY antibody (1:2000). After washing, TMB substrate was added for color development, and the reaction was stopped by adding 1 mol/L H_2_SO_4_. The absorbance was measured at 450 nm using a microplate reader. All assays were performed in triplicate. Error bars represent SDs from triplicate analyses of three independent experiments. Statistical significance was evaluated using a two-way ANOVA in GraphPad Prism 8 software.

Studies have shown that the NDV HN protein can mediate viral entry by binding to α2,3-linked and α2,6-linked sialic acid receptors on the host cell membrane [44,45]. Based on this, receptor-binding activity was measured to determine whether the G347E/E349D dual mutations affect the receptor-binding properties of PPMV-1. As shown in Figure 3, both viruses exhibited binding capabilities to both α-2,3 and α-2,6 sialic acid receptors, with a stronger affinity for α-2,3 receptors compared to α-2,6 receptors. Notably, NT-10-G347E/E349D demonstrated significantly enhanced binding to the α-2,3 sialic acid receptor relative to NT-10 (Figure 3A), while its binding affinity for the α-2,6 receptor was slightly reduced compared to NT-10 (Figure 3B).

**Figure 4:**
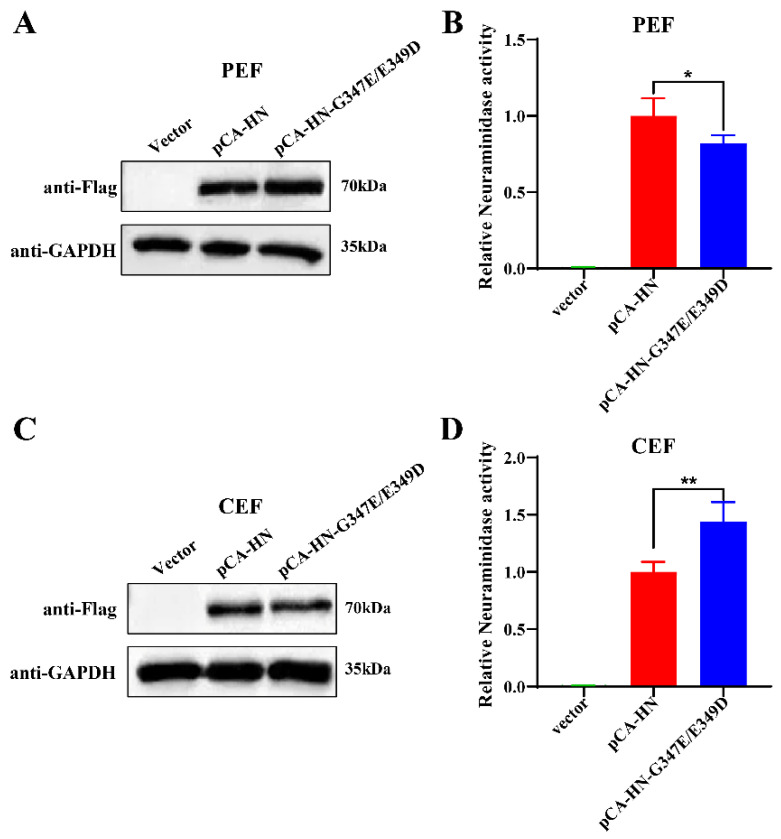
NA activities of HN and G347E/E349D-mutated HN proteins in PEF and CEF cells. PEF and CEF cells were seeded in 6-well plates at a density of 2 × 10⁵ cells per well and transfected with 1 µg of pCA-HN or pCA-HN-G347E/E349D plasmids using the EL Transfection Reagent according to the manufacturer’s protocol. An empty vector (1 µg) was included as a control. At 72 hpt, cells were lysed using RIPA buffer, and HN protein levels in PEF (**A**) and CEF (**C**) cells were measured by Western blot. Neuraminidase activities in PEF (**B**) and CEF (**D**) cells were assessed using a neuraminidase assay kit. The neuraminidase activities were normalized to the values for pCA-HN transfected cells. Error bars represent SDs from triplicate analyses of three independent experiments. Statistical significance was analyzed using one-way ANOVA in GraphPad Prism 8 software. * *p* < 0.05, ** *p* < 0.01. (The original images for blots in Supplementary Figure S1).

In addition to its receptor-binding capability, the NDV HN protein also possesses neuraminidase activity, which degrades sialic acid receptors on progeny virions to prevent self-aggregation, thereby facilitating viral budding [46]. Thus, we further investigated the impact of the G347E/E349D dual mutations on PPMV-1 neuraminidase activity. Given the differences in replication efficiency between NT-10 and NT-10-G347E/E349D in PEF and CEF cells, we transfected PEF and CEF cells with pCA-HN and pCA-HN-G347E/E349D overexpression plasmids. After 72 h, intracellular HN protein levels were measured by Western blot, and neuraminidase activity was assessed using a neuraminidase assay kit. The results showed that both the original and G347E/E349D mutated HN proteins had similar expression levels in PEF (Figure 4A) and CEF (Figure 4C). However, the G347E/E349D-mutated HN protein led to an 18% decrease in neuraminidase activity in PEF cells (Figure 4B) and a 44% increase in CEF cells (Figure 4D) compared to the original HN protein. Overall, these results suggest that the G347E and E349D dual mutations reduce NT-10′s replication and neuraminidase activity in PEF cells while enhancing both in CEF cells. Furthermore, these mutations decrease NT-10′s binding affinity to the α-2,6 sialic acid receptor while significantly increasing its affinity for the α-2,3 receptor.

### 3.5. Evaluation of the Pathogenicity of NT-10 and NT-10-G347E/E349D in Pigeons and Chickens

**Figure 5:**
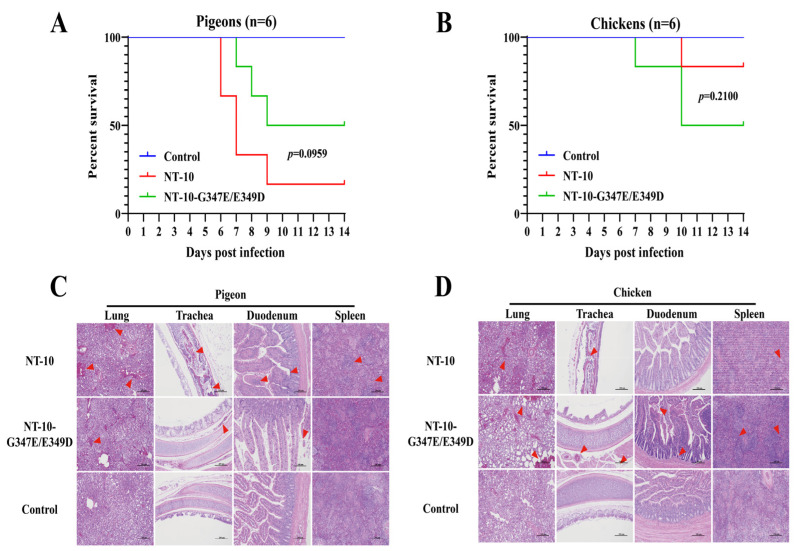
Evaluation of the pathogenicity of NT-10 and NT-10-G347E/E349D in pigeons and chickens. One-month-old White King pigeons (*n* = 9) and four-week-old SPF chickens (*n* = 9) were intranasally infected with 100 μL containing 10⁶ EID50 of either NT-10 or NT-10-G347E/E349D. PBS was used as a control. Six birds in each group were monitored daily for survival over a 14-day period, and survival curves for pigeons (**A**) and chickens (**B**) were generated using GraphPad Prism 8 software. At 5 dpi, three pigeons (**C**) or chickens (**D**) from each group were euthanized, and lung, trachea, duodenum, and spleen tissues were collected for histopathological analysis. The tissues were fixed in 10% neutral-buffered formalin, dehydrated, embedded in paraffin, sectioned, and stained with hematoxylin and eosin (H&E). Lesions, such as inflammatory cell infiltration and tissue damage, are marked with red arrows. Images were captured at 100× magnification with a scale bar representing 200 μm. Statistical significance was analyzed using the Gehan–Breslow–Wilcoxon test in GraphPad Prism software.

To assess whether the G347E and E349D dual mutations affect viral pathogenicity, one-month-old White King pigeons (*n* = 9) and four-week-old SPF chickens (*n* = 9) were infected via intranasal routes with 100 μL containing 10^6^ EID_50_ of either NT-10 or NT-10-G347E/E349D. Six birds in each group were observed daily for survival over a 14-day period. In pigeons, the NT-10 group had two deaths at 6 dpi, two at 7 dpi, and one at 8 dpi, resulting in a 17% survival rate at 14 dpi (Figure 5A). In contrast, the NT-10-G347E/E349D group had one death each at 7, 8, and 9 dpi, with a 50% survival rate at 14 dpi (Figure 5A). In SPF chickens, the NT-10 group only had one death at 10 dpi, yielding an 83% survival rate at 14 dpi, while the NT-10-G347E/E349D group showed increased mortality with deaths at 7 and 10 dpi, resulting in a 50% survival rate at 14 dpi (Figure 5B).

Three birds from each group were euthanized at 5 dpi, and lung, trachea, duodenum, and spleen tissues were collected for histopathological analysis. In pigeons, NT-10 infection induced more severe histopathological damage than NT-10-G347E/E349D, including extensive red blood cell exudation, inflammatory cell infiltration, necrosis, and apoptosis (Figure 5C). In contrast, in SPF chickens, NT-10-G347E/E349D infection caused more severe tissue damage, characterized by extensive alveolar expansion, capillary structural disruption, and inflammatory cell infiltration (Figure 5D). These results collectively indicate that the G347E and E349D mutations reduce the pathogenicity of NT-10 in pigeons while enhancing its pathogenicity in chickens.

### 3.6. Comparison of the Transmission Ability of NT-10 and NT-10-G347E/E349D

**Figure 6:**
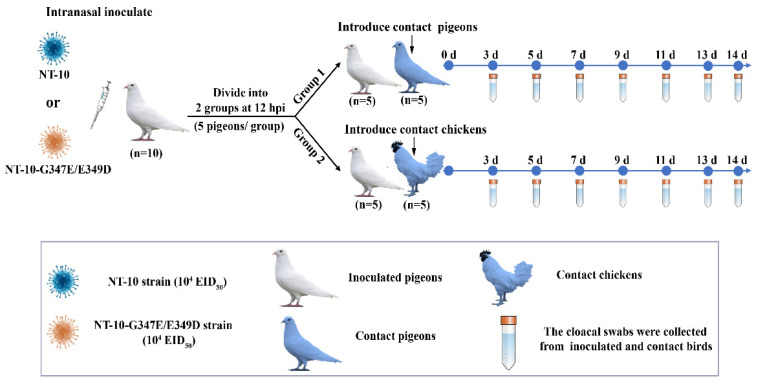
Diagram of the experimental procedure. Briefly, groups of ten White King pigeons were inoculated via intranasal routes with 100 μL containing 10^4^ EID_50_ of either NT-10 or NT-10-G347E/E349D. At 12 hpi, infected pigeons were divided into two groups (5 pigeons per group), and 5 additional pigeons or SPF chickens were placed into the same isolation units for the direct-contact transmission studies. The cloacal swabs for virus shedding detection were collected from inoculated and contact birds at indicated time points. The virus in the collected swabs were detected in the SPF embryonated chicken eggs.

To investigate the impact of the HN protein G347E and E349D dual mutations on the transmissibility of PPMV-1 in pigeons and chickens, one-month-old White King pigeons (*n* = 10) were infected via intranasal routes with 100 μL containing 10^4^ EID_50_ of either NT-10 or NT-10-G347E/E349D. At 12 hpi, infected pigeons were divided into two groups (5 pigeons per group), and five additional pigeons or SPF chickens were placed into the same isolation units for the direct-contact transmission studies. The cloacal swabs were collected from contact birds to monitor viral shedding at indicated time points. A diagram of the experimental procedure is shown in Figure 6.

The viral shedding results were shown in Table 3. In pigeons, viral shedding in the NT-10 contact group began on day 5 and persisted through day 14, with all contact pigeons shedding by that time. In contrast, the NT-10-G347E/E349D contact group first showed viral shedding on day 7, with three birds shedding by day 14. In SPF chickens, only one NT-10 contact bird showed viral shedding, detected on days 11, 13 and 14. Meanwhile, in the NT-10-G347E/E349D contact group, viral shedding was first observed in one bird on day 5 and subsequently detected in four birds by day 14.

To prevent early mortality in the infected pigeons, the inoculation dose was reduced from 10^6^ to 10^4^ EID_50_. Despite this adjustment, one NT-10-infected pigeons died at 13 dpi and 14 dpi, while no deaths were observed in either the NT-10-G347E/E349D-infected or contact pigeons. This outcome further confirms that NT-10-G347E/E349D is less pathogenic in pigeons compared to NT-10. Overall, these results suggest that the G347E and E349D dual mutations reduce the pigeon-to-pigeon transmission of NT-10 but enhance its transmission from pigeons to chickens.

### 3.7. Analysis of Antigenic Differences Between NT-10 and NT-10-G347E/E349D

Amino acids 345–353 have been identified as a linear epitope of NDV HN protein [29,30]. To explore the impact of the G347E and E349D dual mutations on the antigenicity of the PPMV-1 strain, we first analyzed the coefficients of antigenic similarity between NT-10 and NT-10-G347E/E349D using a cross-HI test. As shown in Table 4, a minor antigenic difference was observed between NT-10 and NT-10-G347E/E349D, with an R value of 0.61.

**Figure 7:**
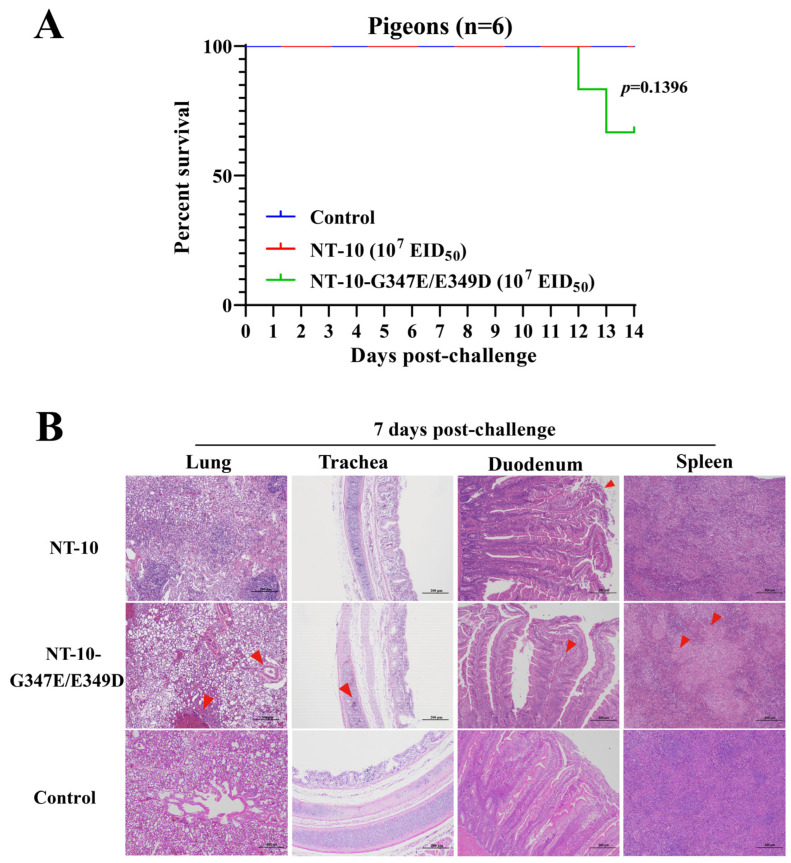
Analysis of antigenic differences between NT-10 and NT-10-G347E/E349D. One-month-old pigeons (*n* = 6 per group) were intramuscularly inoculated with NT-10-based inactivated vaccines at a dose of 10⁴ EID50 per bird. Fourteen days post-vaccination, the pigeons were intranasally challenged with 100 μL containing 10⁷ EID50 of either NT-10 or NT-10-G347E/E349D. PBS was used as a control. Survival was monitored daily for 14 days, and survival curves were generated using GraphPad Prism 8 software (**A**). At 7 dpi, three pigeons from each group were euthanized, and lung, trachea, duodenum, and spleen tissues were collected for histopathological analysis. The tissues were fixed, embedded, sectioned, and stained with H&E. Pathological lesions, such as bleeding, inflammation, and tissue degeneration, are marked with red arrows (**B**). Images were captured at 100× magnification with a scale bar representing 200 μm.

To further confirm the antigenic differences between the mutant virus and its parental strain, NT-10-based inactivated vaccines were prepared and administered intramuscularly to one-month-old pigeons at 10^4^ EID_50_ per bird. Fourteen days post-immunization, the vaccinated pigeons were challenged via intranasal routes with 100 μL containing 10^7^ EID_50_ of either NT-10 or NT-10 G347E/E349D. Six pigeons in each group were observed daily for survival and clinical symptoms over a 14-day period. No deaths or significant clinical symptoms were observed in pigeons challenged with NT-10, indicating that the NT-10 inactivated vaccine provided 100% clinical protection against NT-10 challenge (Figure 7A). However, pigeons challenged with NT-10-G347E/E349D showed a morbidity rate of 50% (3/6) and mortality of 33% (2/6) (Figure 7A), suggesting that the NT-10 inactivated vaccine does not provide complete protection against NT-10-G347E/E349D.

Three pigeons from each group were euthanized at 7 days post-challenge, and tissues were collected for histopathological analysis. As shown in Figure 7B, only minor epithelial shedding of the intestinal villi was observed in the duodenum of NT-10-challenged pigeons. In contrast, pigeons challenged with NT-10-G347E/E349D displayed bleeding, structural changes, and inflammatory cell infiltration in the tissues. These findings collectively indicate that the G347E and E349D dual mutations alter the antigenicity of the NT-10 strain.

## 4. Discussion

Pigeon ND, caused by PPMV-1, is an acute and highly contagious disease, manifesting clinical symptoms similar to those of ND in chickens. PPMV-1 infection leads to high morbidity and mortality, especially in young pigeons, posing significant threats to pigeon farming [47]. First reported in the Middle East in 1977, PPMV-1 quickly spread across Europe, contributing to the third global ND panzootic [14,15]. Today, PPMV-1 remains endemic in several regions, resulting in substantial economic losses in the pigeon industry. Since its introduction to China in the 1980s, PPMV-1 has become established in multiple regions and continues to affect pigeon populations [16,17,18,19,20].

PPMV-1 is recognized as an antigenic variant of chicken-origin NDV, particularly genotype XX NDV, highlighting a close genetic relationship and potential for cross-species transmission [7,8]. Amino acid substitutions in viral proteins may contribute to differences in host range and pathogenicity between PPMV-1 and NDV [9,11,48,49]. However, the precise biological roles of G347E and E349D substitutions in the HN protein remain largely unexplored. In this study, a conservation analysis of 127 PPMV-1 strains and 9 genotype XX NDV strains confirmed the presence of specific substitutions—E347G and D349E—in the HN protein of PPMV-1 compared to genotype XX NDV. These findings suggest potential roles for the HN protein residues at positions 347 and 349 in host adaptation and modulation of virulence. However, the limited availability of genotype XX NDV sequences constrains further exploration of the conservation and functional significance of these residues. Expanded epidemiological studies and increased sequencing efforts are warranted to establish a more comprehensive dataset for analyzing the conservation of amino acids 347 and 349 in the HN protein.

To investigate the role of amino acids at positions 347 and 349 in the HN protein, we compared the infection and transmission abilities of three PPMV-1 strains. Interestingly, although all three PPMV-1 strains share the same F protein cleavage site motif (^112^RRQKRF^117^), their pathogenicity in pigeons varied. This observation reinforces the notion that the F protein cleavage site is not the sole determinant of NDV virulence. Given that NT-10 shares 94% and 92% amino acid homology with the other two PPMV-1 strains, we hypothesize that NT-10′s higher virulence may result from genetic variations in other regions of the genome. These variations likely enhance NT-10′s replication efficiency in pigeons, contributing to its increased pathogenicity and transmission capabilities.

The HN protein serves multiple functions, including receptor recognition and receptor removal on progeny virions to prevent self-aggregation [46]. In this study, we found that the G347E and E349D mutations significantly reduced NT-10′s replication efficiency in PEF cells but enhanced it in CEF cells. Receptor-binding assays revealed that NT-10-G347E/E349D had a higher affinity for α-2,3 sialic acid receptors and a lower affinity for α-2,6 receptors compared to NT-10. This change in receptor preference likely accounts for NT-10-G347E/E349D’s increased replication in CEF cells and reduced efficiency in PEF cells. Studies have shown that pigeons predominantly express α-2,6 sialic acid receptors in their respiratory tract, whereas chickens have a higher abundance of α-2,3 receptors [50]. Such receptor-binding changes are crucial for cross-species transmission in viruses, as demonstrated in avian influenza viruses, where an increased affinity for α-2,6 receptors is associated with a higher risk of zoonotic transmission [51,52]. Thus, it is plausible that the G347E and E349D mutations represent adaptive changes acquired by genotype XX NDV in pigeons, leading to the emergence of PPMV-1 with enhanced replication in pigeon hosts.

Our neuraminidase activity assays demonstrated that NT-10 exhibited higher neuraminidase activity in PEF cells, whereas NT-10-G347E/E349D showed increased activity in CEF cells. Neuraminidase activity is essential for efficient viral release by preventing virion aggregation on the cell surface, thus facilitating viral budding and dissemination [53]. Enhanced neuraminidase activity is often linked to increased virulence and replication efficiency [54]. The elevated neuraminidase activity of NT-10 in PEF cells likely contributes to its enhanced replication and potential transmissibility in pigeons, reinforcing its adaptation to this host.

Pathogenicity assessments further revealed that the G347E and E349D mutations shift virulence patterns across hosts. Consistent with previous reports, PPMV-1 typically shows high pathogenicity in pigeons, with limited clinical signs in other domestic birds such as chickens [7,55,56,57]. In our study, NT-10 displayed high mortality in pigeons (17% survival), while infected chickens had a survival rate of 83%, confirming PPMV-1′s higher virulence in pigeons. Notably, NT-10-G347E/E349D caused equal survival rates (50%) in both pigeons and chickens, indicating that these mutations reduce NT-10′s host specificity for pigeons while increasing its pathogenicity in chickens. A statistical analysis of the survival curves, however, showed no statistically significant difference in survival rates between NT-10 and NT-10-G347E/E349D in either species under the conditions tested. We attribute this to the limited sample size (*n* = 6 per group), which likely reduced the statistical power to detect subtle but biologically meaningful differences. Despite the lack of statistical significance, the observed trends in survival rates strongly suggest that residues G347 and E349 in the HN protein are critical determinants of PPMV-1′s host specificity.

PPMV-1 is known to efficiently spread within pigeon populations but not among chickens or ducks [56,57]. Consistent with these findings, all contact pigeons in the NT-10 group showed viral shedding within 14 days, whereas only one contact chicken exhibited shedding. However, in the NT-10-G347E/E349D group, 60% of contact pigeons and 80% of contact chickens showed viral shedding, indicating that the G347E and E349D mutations enhance NT-10′s transmissibility to chickens while reducing its pigeon-to-pigeon transmission efficiency. These findings further highlight the importance of residues 347 and 349 in PPMV-1′s efficient transmission within pigeon hosts.

In addition to mediating viral binding and facilitating viral release, the HN protein is the primary antigenic component of NDV. The region encompassing amino acids 345–353 contains the only known linear epitope on the HN protein, which is also one of the most variable antigenic sites [29,30]. Studies have shown that vaccines based on chicken-origin NDV strains, such as LaSota, do not fully protect pigeons against PPMV-1 challenges, suggesting antigenic differences between PPMV-1 and chicken-origin NDV [34,58,59]. In this study, we found that NT-10-based inactivated vaccines provided full protection against NT-10 but only partial protection against NT-10-G347E/E349D, with 50% morbidity and 33% mortality in NT-10-G347E/E349D-challenged pigeons. This result suggests that amino acid differences at positions 347 and 349 contribute to the observed antigenic variation between PPMV-1 and chicken-origin NDV strains.

In summary, this study highlights the multifaceted role of amino acids at positions 347 and 349 in the HN protein in determining PPMV-1′s host specificity, pathogenicity, transmission efficiency, and antigenicity. These findings deepen our understanding of the molecular mechanisms underlying PPMV-1 pathogenesis and cross-species transmission, providing a basis for developing targeted control measures, including updated vaccines and improved biosecurity strategies, to mitigate the impact of PPMV-1 in poultry farming.

## Figures and Tables

**Table 1:** Comparison of pathogenicity and transmission abilities in pigeons among three PPMV-1 Strains.

Virus	Group	Dose	Days Post Infection (dpi)						
			3	5	7	9	11	13	14
NT-10	Infected	10^6^ EID_50_	3/6 ^a^	5/6	4/4 ^b^	2/2 ^b^	1/1 ^b^	1/1	1/1
	Contact	-	0/4	1/4	3/4	3/4	4/4	4/4	4/4
JS/09/16/Pi	Infected	10^6^ EID_50_	3/6	3/6	4/6	3/4 ^b^	2/3 ^b^	2/3	2/3
	Contact	-	0/4	0/4	1/4	1/4	2/4	2/4	2/4
JS/07/04/Pi	Infected	10^6^ EID_50_	3/6	3/6	3/5 ^b^	3/4 ^b^	3/4	2/3 ^b^	2/3
	Contact	-	0/4	0/4	1/4	1/4	2/4	2/4	2/4
PBS	Infected	-	0/6	0/6	0/6	0/6	0/6	0/6	0/6
	Contact	-	0/4	0/4	0/4	0/4	0/4	0/4	0/4

^a^ Number of viral shedding pigeons/total number of pigeons. ^b^ Mortality among pigeons led to a reduction in the number of viral shedding pigeons and total number of pigeons.

**Table 2:** The biological characteristics of NT-10 and NT-10-G347E/E349D.

Virus	Biological Characteristics		
	EID_50_/0.1 mL ^a^	MDT (h) ^b^	ICPI ^c^
NT-10	10^7.0^	69	1.34
NT-10-G347E/E349D	10^7.83^	58	1.51

^a^ The 50% infectious dose in SPF chicken embryo. ^b^ Mean death time in SPF chicken embryo (h) (<60, velogenic; 60–90, mesogenic; >90, lentogenic). ^c^ Intracerebral pathogenicity index in day-old SPF chicks (1.4–2.0, velogenic; 0.7–1.4, mesogenic; <0.7, lentogenic).

**Table 3:** Comparison of the transmission ability of NT-10 and NT-10-G347E/E349D.

Bird	Virus	Dose	Days Post Infection						
			3 d	5 d	7 d	9 d	11 d	13 d	14 d
Inoculated pigeons	NT-10	10^4^ EID_50_	2/5 ^a^	3/5	4/5	5/5	5/5	4/4 ^b^	3/3 ^b^
	NT-10-G347E/E349D	10^4^ EID_50_	0/5	1/5	2/5	3/5	3/5	4/5	4/5
Contact pigeons	NT-10	-	0/5	1/5	2/5	3/5	3/5	4/5	5/5
	NT-10-G347E/E349D	-	0/5	0/5	1/5	1/5	1/5	2/5	3/5
Inoculated chickens	NT-10	10^4^ EID_50_	0/5	1/5	1/5	2/5	3/5	3/5	4/5
	NT-10-G347E/E349D	10^4^ EID_50_	1/5	2/5	3/5	4/5	4/5	5/5	5/5
Contact chickens	NT-10	-	0/5	0/5	0/5	0/5	1/5	1/5	1/5
	NT-10-G347E/E349D	-	0/5	1/5	2/5	2/5	3/5	3/5	4/5

^a^ Number of viral shedding birds/total number of birds. ^b^ Mortality among pigeons led to a reduction in the number of viral shedding birds and total number of birds.

**Table 4:** Coefficients of antigenic similarity between NT-10 and NT-10-G347E/E349D.

Virus	R Value ^a^ with NT-10
NT-10	1.00
NT-10-G347E/E349D	0.61

^a^ 0.67 ≤ R ≤ 1.5 indicates no significant antigenic difference between the two viruses; 0.5 ≤ R ≤ 0.67 indicates a minor difference between the two viruses; and R < 0.5 indicates a major difference between the two virus strains.

## Data Availability

The data presented in this study are available upon request from the corresponding authors.

## Supplementary Materials

The following supporting information can be downloaded at: https://www.mdpi.com/article/10.3390/vetsci11120592/s1, Figure S1: Original Images for Blots (Figure 4A,C).
