# Supplementary figures and images for: The Role of Dual Mutations G347E and E349D of the Pigeon Paramyxovirus Type 1 Hemagglutinin–Neuraminidase Protein In Vitro and In Vivo
Source: Vet Sci. 2024 Nov 25;11(12):592. doi: 10.3390/vetsci11120592 (PMC11680095; doi:10.3390/vetsci11120592)

Figure 4A

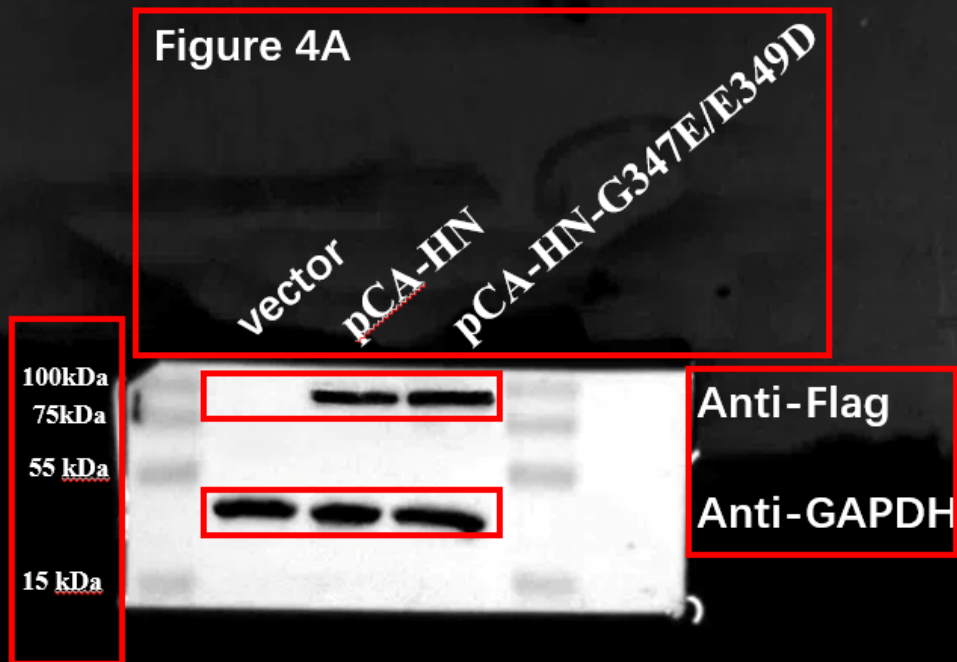

Figure 4C

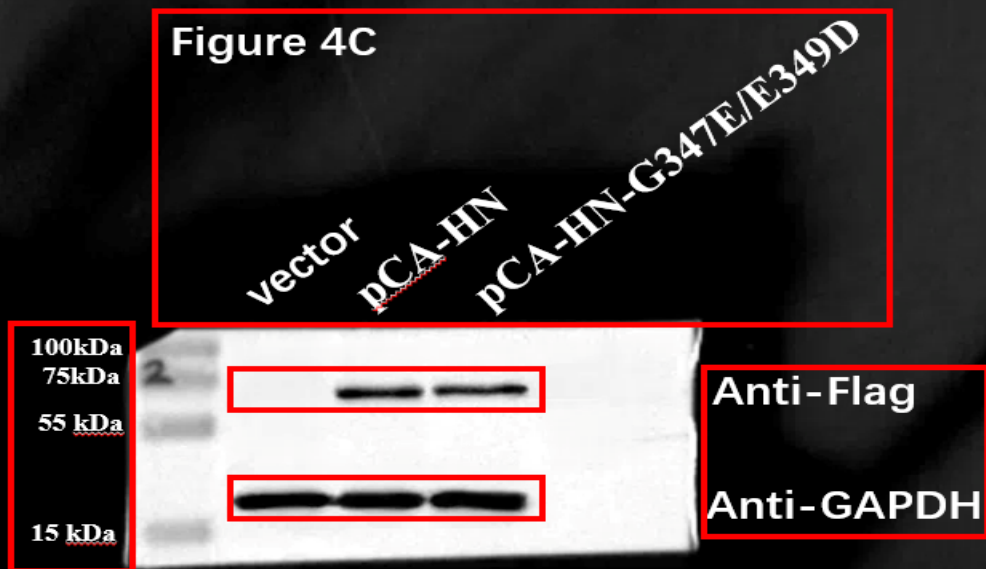

Supplement: Supplementary file 1 [file vetsci-11-00592-s001.zip › vetsci-3317931-supplementary.pdf]
